# Intermittent and Short‐Term Empirical Ruxolitinib Regimen for Steroid‐Refractory Flareups of Fibrodysplasia Ossificans Progressiva

**DOI:** 10.1002/prp2.70193

**Published:** 2025-11-14

**Authors:** Rong‐Long Chen, Tzu‐Hsien Yang, Yun‐Hsin Wang, Jy‐juinn Shaw, Liuh‐Yow Chen

**Affiliations:** ^1^ Department of Pediatric Hematology and Oncology Koo Foundation Sun Yat‐Sen Cancer Center Taipei Taiwan; ^2^ Institute of Molecular Biology, Academia Sinica Taipei Taiwan; ^3^ Department of Molecular Medicine Koo Foundation Sun Yat‐Sen Cancer Center Taipei Taiwan

**Keywords:** fibrodysplasia ossificans progressiva, flareup, heterotopic ossification, human activin receptor type I, Janus‐associated kinase inhibitor, ruxolitinib

## Abstract

Fibrodysplasia ossificans progressiva (FOP) is an ultra‐rare genetic disorder with inflammation‐related flare‐ups resulting in catastrophic heterotopic ossification (HO). Janus‐associated kinase (JAK) inhibitors may have had a blocking effect on bone formation in controlling FOP flare‐ups by blocking multiple inflammatory signaling pathways. Continuous JAK inhibitor tofacitinib treatment has shown preliminary safety and effect in preventing FOP flare‐ups. There is concern that the use of long‐term continuous JAK inhibitors might cause renal, hepatic, and hematological toxicity, as well as increased infections and cancers. We incorporated a six‐week ruxolitinib (another JAK inhibitor) regimen given intermittently for empirical use at the flare‐up onset in a teenager after she experienced three consecutive corticosteroid‐refractory severe lower limb FOP flare‐ups within 1 year. The regimen proved well‐tolerated with efficacy in terms of blocking morbidity‐generating heterotopic ossification and extending the flare‐up‐free intervals to 5, 12, and 36 months until subsequent flare‐ups, respectively, accompanied by a stable cumulative analog joint involvement scale (CAJIS) score for the subsequent 5 years. The regimen appeared to inhibit new bone formation and may avoid long‐term use‐related toxicities in FOP patients.

**Abbreviations**
ACVR1: activin receptor type I
CAJIS: cumulative analog joint involvement scale
FOP: Fibrodysplasia ossificans progressiva
GVHD: graft versus host disease
HO: heterotopic ossification
JAK: Janus‐associated kinase
NASID: non‐steroidal anti‐inflammatory drug

## 1 Introduction

Fibrodysplasia ossificans progressiva (FOP, MIM 135100) is a rare genetic disorder most commonly caused by a heterozygous c.617G>A (Arg206His) germline missense mutation in the *ACVR1* (human activin receptor type I) gene, leading to specific anatomical patterns of progressive heterotopic ossification (HO) and cumulative immobility [1]. FOP flareups, progressively catastrophic events that result in severe patient immobility, are attributable to immune hyperactivation of various local inflammatory agents triggered by diverse insults, which induce tissue inflammation, fibrosis, endochondral ossification, and ultimately, aberrant and HO‐linked morbidity [2]. Early administration of a high‐dose corticosteroid plus a non‐steroidal anti‐inflammatory drug (NSAID) at symptom onset of critical flareups has been widely deployed as a class I treatment [3]. Novel drugs that specifically target ACVR1 are actively being developed, but they are currently not available for general use [3]. For example, garetosmab, a monoclonal antibody against activin A, was recently demonstrated to reduce the HO lesions formed during FOP flareups in adults, but its benefit–risk profile requires further evaluation [4]. Ruxolitinib, an orally administered Janus‐associated kinase (JAK) 1/2 inhibitor, has been applied successfully in treating various pediatric immune hyperactivation and inflammatory disorders such as steroid‐refractory pediatric graft versus host disease (GVHD) [5] and hemophagocytic lymphohistiocytosis [6]. Nikishina et al. have recently reported their observations on the efficacy of a continuous regimen with tofacitinib, which involved administering 5 mg of this JAK inhibitor twice a day to 13 patients with FOP refractory to standard of care treatment [7]. Given the promising results of ruxolitinib treatment in children having other inflammatory disorders and the fact that it has displayed efficacy in blocking neurogenic HO in a preliminary mouse model [8], we repurposed the preemptive ruxolitinib regimen in an emergency off‐label use to treat and prevent flareups in an FOP patient to prevent HO morbidity.

## 2 Patient and Method

**FIGURE 1:**
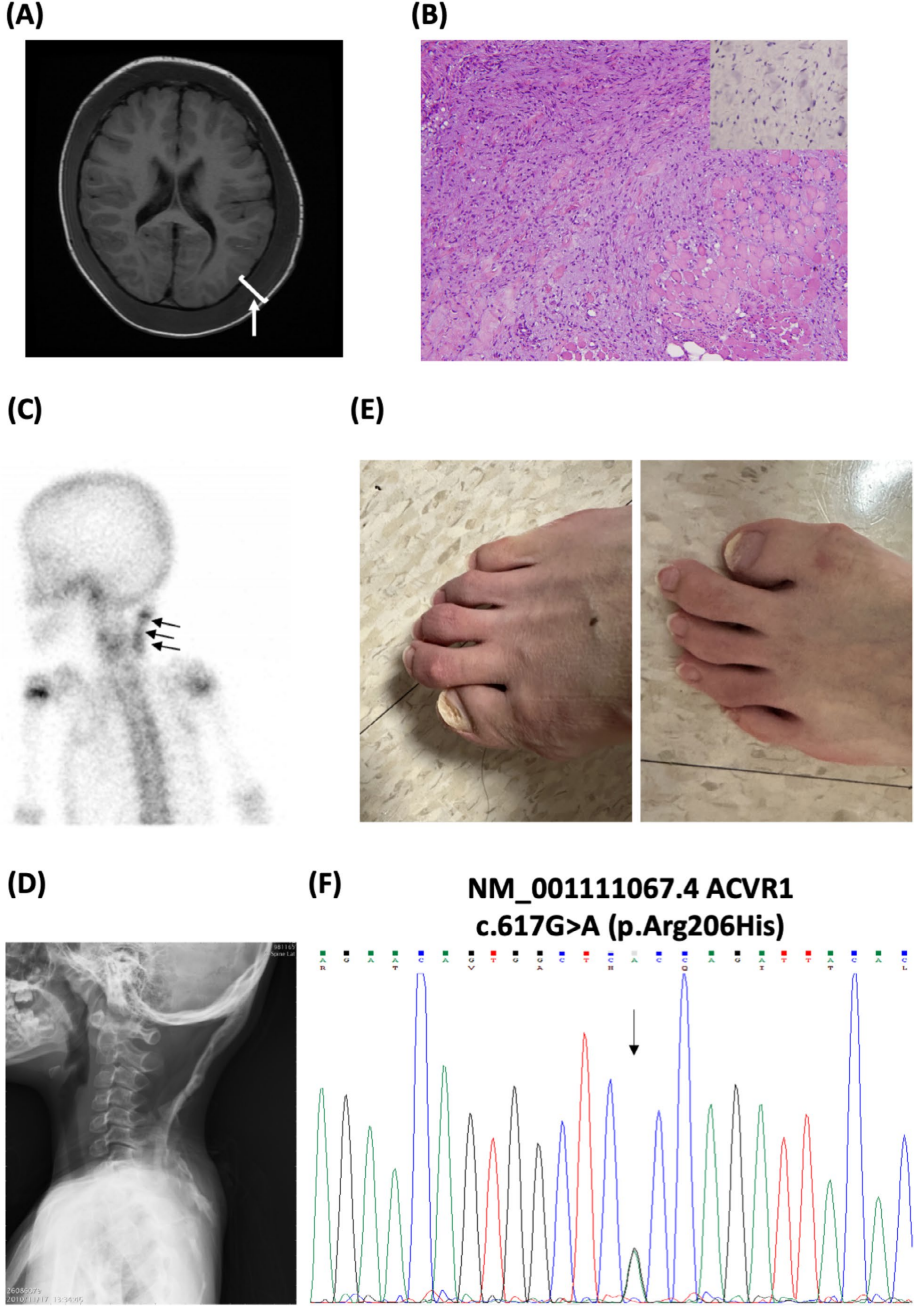
(A) Pre‐contrast magnetic resonance imaging of a T1 axial view showing a diffuse soft tissue lesion along the subgaleal space involving the whole calvarium and spreading downward to the neck via the aponeurosis space (arrow and bracket) at presentation of the 2.5‐year‐old female patient. (B) Standard hematoxylin and eosin staining from posterior neck nodule biopsy at diagnosis, showing diffuse spindle cell proliferation with negative beta‐catenin immunohistochemical staining (insert). (C) Tc^99m^ bone scan performed ~1.5 months after nape biopsy, revealing active heterotopic ossification over the biopsy site. (D) Development of extensive linear calcification/osteogenesis over the posterior neck and extending to the left shoulder, causing functional ankylosis. (E) Characteristic features of bilateral great toe microdactyly and hallux valgus of the patient. (F) Sanger DNA sequencing of *ACVR1* exon 6 on chromosome 2 from peripheral blood cells taken from the patient.

The female patient initially presented with multiple lumps over her scalp and left periorbital soft tissues after a febrile respiratory infection when she was 2.5 years of age. A course of antibiotics was administered under the impression of a cellulitis diagnosis. However, the scalp masses grew rapidly, extended to the posterior nape, and resulted in diffuse enlargement of the cranial vault (Figure 1A). As the lesions progressed, a biopsy from a posterior cervical lump was taken, with pathology revealing a primitive spindle cell proliferative lesion for which immunohistochemistry was positive for smooth muscle actin, but negative for CD34 and beta‐catenin (Figure 1B). The patient was then transferred to our care. The cranial lesions began to contract spontaneously, and the scalp lesions resolved completely 1 week after biopsy, with HO appearing over the biopsy site (Figure 1C). The biopsy‐site lesion gradually extended, resulting in extensive linear ossification over the posterior neck (Figure 1D), as well as ankylosis of the neck and left shoulder girdle. FOP was initially diagnosed on clinical grounds including the congenital presence of bilateral great toe microdactyly and hallux valgus (Figure 1E) and confirmed by the identification of a pathogenic missense mutation of *ACVR1* at codon 206 (c.617G>A, R206H) (Figure 1F).

**FIGURE 2:**
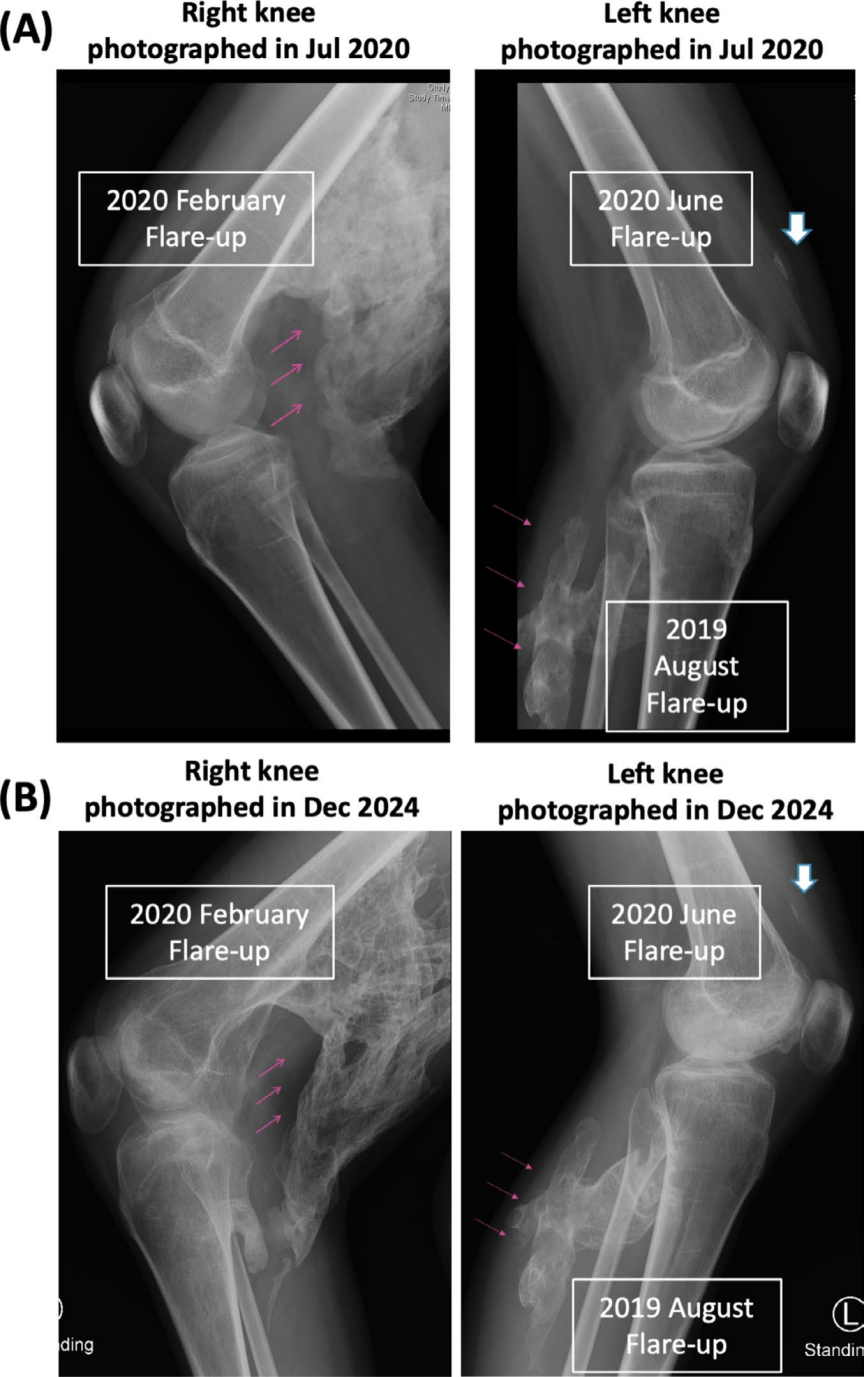
Illustrative knee X‐rays post‐ruxolitinib treatment initiation taken in July 2020 (1 month after initiation of the ruxolitinib regimen for a flareup of the left knee upper soft tissue) (A) and December 2024 (4.5 years later) (B). Severe heterotopic ossification over the left calf and right knee (red arrows) resulting in right knee ankylosis, but the flareup of the left knee upper soft tissue only resulted in trace calcification (white arrows). The degree of right knee heterotopic ossification (red arrows) slightly decreased in December 2024 compared to July 2020.

To assess the functional mobility of our FOP patient, we applied the cumulative analog joint involvement scale (CAJIS) scoring system [9]. In this system, each joint is scored as 0 (normal), 2 (functionally ankylosed), or 1 (mobility restriction between 0 and 2). The evaluation includes three axial joints (neck, thoraco‐lumbar spine, jaw), as well as three joints from each of the four limbs (shoulder, elbow, and wrist for the upper limbs; hip, knee, and ankle for the lower limbs), yielding a total score ranging from 0 to 30 at each assessment. She was educated and placed on a comprehensive FOP treatment guideline, including intermittent short‐term high‐dose prednisolone (2 mg/kg daily for 3–5 days for critical flareups defined by rapid painful swellings of appendicular or submandibular attacks) with or without other agents (ibuprofen, montelukast, baclofen). The regimen initially worked well and the CAJIS score remained 5, with functional ankylosis in the neck and left shoulder, as well as a mildly affected thoracic spine, until the age of 13 years. A painful posterior swelling (flareup) in the left leg arose in the middle of 2019 when she was 13.5 years of age. She was administered high‐dose prednisolone (80 mg daily) for 4 days as usual, but the swelling persisted for more than 2 weeks and resulted in severe HO over the left calf (Figure 2). The function of her left knee was unaffected. At 14 years of age (February 2020), another painful flareup arose over the right buttock and posterior thigh, with severe HO extension despite repeated intermittent high‐dose prednisolone over the course of 2 months. Extended HO resulted in ankylosis of the right hip and knee (Figure 2) and it functionally affected the left hip. The CAJIS score abruptly increased to 11, and she shifted from being walk‐free to requiring a wheelchair. She also suffered from steroid‐related Cushing syndrome and displayed excess weight gain (from ~40 to > 50 kg).

At this timepoint (early 2020), we successfully applied for Institute Review Board (IRB) approval for an off‐label re‐purposing of an empirical ruxolitinib regimen (Table 1) with the written informed consent of the FOP patient and her mother, and the study was conducted according to the Declaration of Helsinki. Only months later, when she was ~14.5 years of age (June 2020), another painful flareup occurred over the soft tissue above the left knee.

**TABLE 1:** Empirical ruxolitinib regimen for FOP flareups.

Schedule	Initial cycle	Subsequent cycles
Escalation (Day −6 to 0)	Ruxolitinib 5 mg PO^a^ BID^b^	None
Day 1 to 14	Ruxolitinib 10 mg PO^a^ BID^b^	Ruxolitinib 10 mg PO^a^ BID^b^
Day 15 to 28	Ruxolitinib 5 mg PO^a^ BID^b^	Ruxolitinib 5 mg PO^a^ BID^b^
Day 29 to 42	Ruxolitinib 5 mg PO^a^ QD^c^	Ruxolitinib 5 mg PO^a^ QD^c^

^a^ PO: per os.

^b^ BID: twice‐daily dosage.

^c^ QD: once a day.

## 3 Results

After 2 days of high‐dose prednisolone, which did not stall progression of the painful swelling, we applied IRB‐approved off‐label re‐purposing of ruxolitinib with the written informed consent of the patient and her mother, starting from 5 mg twice daily for 1 week that was escalated to 10 mg twice daily for another 2 weeks based on a body weight at the time (June 2020) of 52.2 kg. Thereafter, we administered 5 mg of ruxolitinib twice daily for 2 weeks, followed by 5 mg daily for a further 2 weeks. This regimen not only blocked new HO development over the left knee (Figure 2), but also ameliorated the swelling of the right thigh that had persisted for months. Subsequently, she had three further flare‐ups (the right scapula in November 2020, the right mandibula in November 2021, and the lower chin in November 2024) without consequences after undergoing the 6‐week ruxolitinib regimen for the subsequent cycles (Table 1) over the next 5 years. No NSAIDs nor corticosteroids were given, and her body weight reverted back to her baseline of 43 kg. There were no serious adverse events based on periodic clinical and laboratory evaluations, including myelotoxicities or infections, throughout the entire treatment course. She is now a 19‐year‐old university student. The CAJIS score remains at 11, that is, the same as that before initiating the ruxolitinib regimen.

## 4 Discussion

A majority of FOP patients suffer flareups that result in progressive functional impairment, despite short‐term high‐dose steroid treatment [3]. There is a clear age‐related pattern of limb swelling among FOP patients, with lower limb flareups more commonly occurring at a later age and becoming chronic relative to upper limb flareups [10]. FOP pathogenesis has been attributed to novel functions of the ACVR1 R206H pathogenic variant related to neoligand Activin A‐induced receptor clustering and autophosphorylation [11]. However, how that exacerbates inflammation of extra‐skeletal tissues and how the inflammation by many types of agents leads to osteogenic differentiation only remains partially understood. Nevertheless, ruxolitinib treatment has proven effective in reducing neurogenic HO volume and STAT3 phosphorylation in the injured muscles of a mouse model [2, 8]. Several repurposed medications targeting specific pathogenic mutations linked to FOP have been identified [3]. Since *ACVR1*‐mutation‐specific medications are currently not available, we re‐purposed ruxolitinib given the emergency status of our patient. Although a controlled clinical trial would be needed to assess the efficacy of any drug for FOP due to the episodic nature and spontaneous resolution of flareups, our preliminary results of a preemptive ruxolitinib regimen for managing FOP flareups in this case are promising. As long‐term ruxolitinib use might cause renal, hepatic, and hematological toxicity and have a peculiar immunosuppressive effect (with an increased incidence of infections and occurrence of nonmelanoma skin tumors/aggressive lymphomas) [12], we adopted an intermittent and short‐term empirical regimen, thereby endeavoring to exert an osteogenetic blocking effect while avoiding severe toxicity. The patient had received three additional courses of ruxolitinib for flareups during the 5‐year follow‐up period with a stable CAJIS score and no apparent adverse effects according to periodic clinical and laboratory evaluations. As with our experience described herein, a recent phase II trial of ruxolitinib for the treatment of steroid‐refractory chronic GVHD indicated that, overall, it was well tolerated and prompted relatively high rates of skin/joint responses [12]. In conclusion, we present a successful case of emergency salvage of a patient with critical lower limb FOP flareups that, otherwise, could have resulted in much more severe morbidity.

## Author Contributions

R.‐L.C.: conceptualization, data curation, funding acquisition, investigation, project administration, writing – original draft. T.‐H.Y.: data curation, investigation, resources, writing – review and editing. Y.‐H.W.: data curation, investigation, resources. J.‐j.S.: funding acquisition, project administration, supervision, writing – review and editing. L.‐Y.C.: funding acquisition, resources, supervision, writing – review and editing.

## Disclosure

Principal investigator statement: The authors confirm that the Principal Investigator for this paper is Dr. Rong‐Long Chen and that he had direct clinical responsibility for patients.

## Ethics Statement

The off‐label re‐purposing of the ruxolitinib regimen has been approved by the Institute Review Board (IRB) of the Koo Foundation Sun Yat‐Sen Cancer Center.

## Consent

We have obtained the written informed consent from the patient and her mother.

## Conflicts of Interest

The authors declare no conflicts of interest.

## Data Availability

Nomenclature of targets and ligands: Key protein targets and ligands in this article are hyperlinked to corresponding entries in http://www.guidetopharmacology.org, the common portal for data from the IUPHAR/BPS Guide to PHARMACOLOGY [13], and are permanently archived in the Concise Guide to PHARMACOLOGY 2023/24 [14, 15].
